# Massive Accumulation of Glycosaminoglycans in the Aortic Valve of a Patient With Hunter Syndrome During Enzyme Replacement Therapy

**DOI:** 10.1007/s00246-013-0653-0

**Published:** 2013-02-13

**Authors:** Yohei Sato, Masako Fujiwara, Hiroshi Kobayashi, Hiroyuki Ida

**Affiliations:** Department of Pediatrics, The Jikei University School of Medicine, 3-25-8 Nishi-shimbashi, Minato-ku, Tokyo, Japan

**Keywords:** Aortic valve replacement, Enzyme replacement therapy, Glycosaminoglycan, Hunter syndrome, Mucopolysaccharidosis

## Abstract

This report describes the pathologic findings for a patient with Hunter syndrome who underwent aortic valve replacement at 10 years of age, 3 years after the initiation of enzyme replacement therapy. Aortic valve pathology showed mild thickening and fibrosis as well as massive glycosaminoglycan accumulation. This suggests that enzyme replacement therapy has limited efficacy for cardiac valve disease both clinically and pathologically.

Hunter syndrome, mucopolysaccharidosis type 2, is an X-linked lysosomal storage disorder caused by an iduronate-2-sulfatase deficiency. The accumulation of glycosaminoglycans (GAGs) causes progressive systemic organ failure. Major mortality factors are central nervous system involvement, cardiac involvement, and upper airway obstruction. Death in the first or second decade of life is common among severely affected patients [8].

Enzyme replacement therapy (ERT) with recombinant human iduronate-2-sulfatase (idursulfase) improves clinical manifestations, such as dyspnea and abnormal gait, by reducing the accumulation of GAGs in lysosomes [5, 6]. Because almost all patients with Hunter syndrome experience the development of cardiac abnormalities, cardiac evaluations, including 12-lead electrocardiography and echocardiography, are recommended every 1–3 years [7, 9].

However, it still is unknown whether ERT actually can ameliorate or prevent cardiac valve disease in Hunter syndrome. We report a patient with Hunter syndrome receiving ERT who underwent aortic valve replacement surgery.

## Case Report

A 3-year-old boy was referred for progressive neurologic deterioration. Hunter syndrome was confirmed by a deficiency of iduronate-2-sulfatase enzyme activity. Annual cardiac follow-up assessment, including electrocardiography and echocardiography, was performed, and the boy was found to have aortic regurgitation (AR) at the age of 5 years.

When the boy was 7 years old, ERT with idursulfase (Elaprase) was initiated, but immunoglobulin-G (IgG) antibody developed during ERT. The antibody titer was negative at the initiation of ERT, then found to be 1:80 at 15 months, 1:800 at 20 months, and 1:1,600, at 24 months after the initiation of ERT. Aortic regurgitation and left ventricular (LV) dysfunction progressed despite the ERT. Cardiac catheterization and angiography showed an increase in LV end-diastolic volume and a progression of AR.

The patient underwent aortic valve replacement (AVR) 3 years after the initiation of ERT. A 17-mm Regent prosthetic aortic valve (St. Jude Medical Inc., St. Paul, MN, USA) was implanted. The aortic valve was sent to the pathology department for analysis. Throughout surgery for AVR, ERT was continued, even during the perioperative period.

**Fig. 1:**
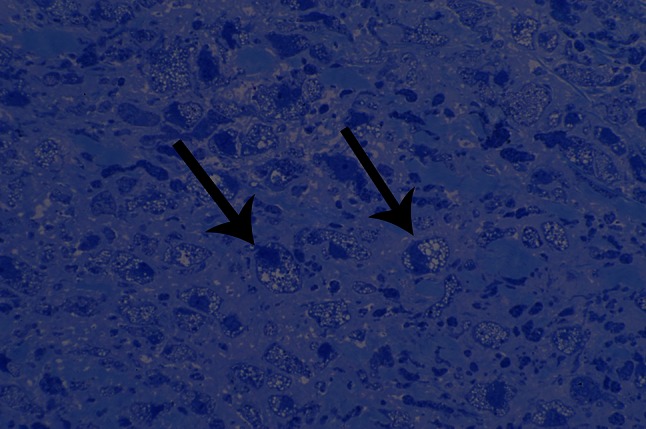
General pathology of the aortic valve. Toluidine *blue* stain shows lysosomal accumulation of glycosaminoglycans

Hematoxylin and eosin (H&E) staining showed mild thickening and interstitial fibrosis of the aortic valve. We also observed vacuolated histiocyte infiltration, otherwise known as “clear” cells. Toluidine blue staining of the aortic valve demonstrated cellular accumulation of GAGs (Fig. 1).

**Fig. 2:**
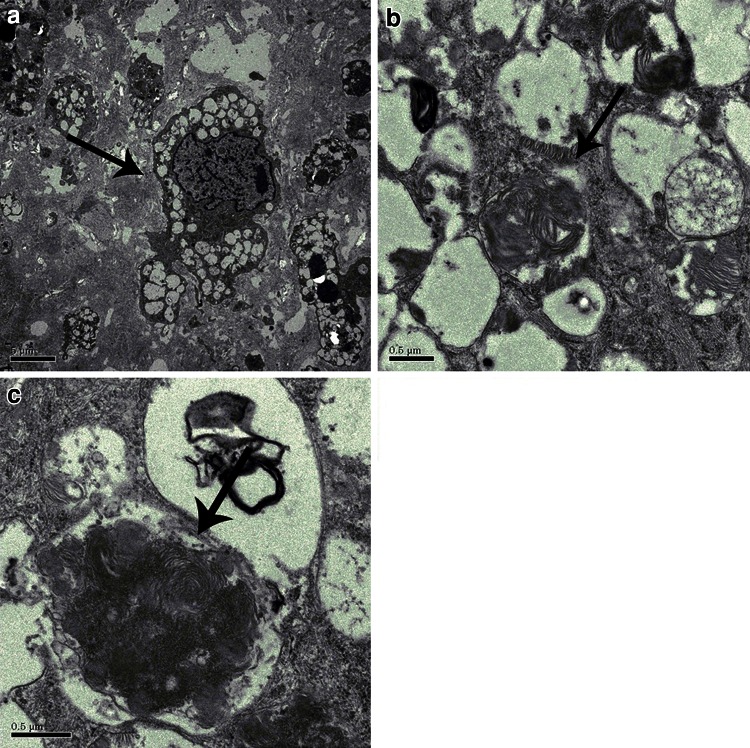
Electron microscopy of the aortic valve. **a** *Arrow* shows a vacuolated histiocyte, indicating lysosomal accumulation of glycosaminoglycans. **b,** **c** In the lysosomes, zebra body formation indicating lysosomal accumulation of glycosaminoglycans can be seen

Electron microscopy examination of the aortic valve showed GAG-laden cells, similar to the general pathology finding. In addition, we observed zebra body formation, which indicated glycolipid accumulation (Fig. 2).

The patient continued to receive ERT 2 years after surgery and was asymptomatic. Follow-up cardiac echocardiography showed a remarkable improvement in AR and LV function.

## Discussion

Hunter syndrome manifests with progressive systemic organ failure due to GAG accumulation caused by the lack of iduronate-2-sulfatease. Muenzer et al. [5, 6] showed that ERT is associated with a reduction in urine GAG levels and organ size, as well as with an increased 6-min walk test distance.

Cardiac involvement is common in patients with Hunter syndrome. Wraith et al. [8] reported that 57 % of patients with Hunter syndrome have cardiac valve disease. Mitral and aortic valves are more likely to be affected than right-sided heart valves. Some reports have shown that patients with Hunter syndrome require regular follow-up assessment, such as electrocardiography and cardiac echocardiography, every 1–3 years [7, 9].

Before the development of ERT, some surgical cases of mucopolysaccharidosis were reported for valve replacement surgery. Bhattacharya et al. [2] and Antoniou et al. [1] reported mitral valve replacement surgery for mitral stenosis secondary to Hunter syndrome. Both patients in these previous reports were adults who had never received ERT.

Cardiac pathology of mucopolysaccharidosis has been studied more in type 1 than in type 2. Mucopolysaccharidosis type 1 cardiac valve pathology characteristically shows increased GAG content as well as infiltration of “clear” cells (Hurler cells) [3]. The reported patient had the pathologic finding of mucopolysaccharidosis type 2, similar to type 1, in the aortic valve. It has been suggested that Hurler syndrome and Hunter syndrome have a similar pathophysiology of cardiac valve disease.

Fesslova et al. [4] showed progression of cardiac valve disease in a patient with mucopolysaccharidosis types 1 and 2. In their study of eight patients who had cardiac valve disease and were receiving ERT, four patients had progressive disease, and the remaining four patients had stable disease.

For the reported patient, ERT was initiated after AR had already developed. Regardless of the ERT, cardiac valve disease deteriorated, and finally, AVR was performed. The finding of GAG accumulation in the aortic valve of the reported patient suggests that ERT does not reverse cardiac valve disease in Hunter syndrome. Therefore, ERT might not be effective after the onset of cardiac valve disease. Valve replacement surgery for a patient with Hunter syndrome during ERT has not been previously reported.

The actual mechanism of cardiac disease in mucopolysaccharidosis remains unknown. The heparin-, dermatan-, chondroitin-, and keratin-sulfate GAGs are normal components of cardiac valves. Accumulation of GAG is the main pathology of cardiac involvement in mucopolysaccharidosis, resulting in hemodynamic decongements [3].

We report, for the first time, AVR surgery for a patient with Hunter syndrome during ERT. In the reported patient, although mild cardiac valve disease had developed at the initiation of ERT, it was refractory to a 3-year course of ERT. This suggests that ERT has a limited efficacy for cardiac valve disease both clinically and pathologically. Antibody formation could hinder the effects of ERT. We hypothesize that idursulfase does not reach the cardiac valve because of its poor vascularity. Further study is required to determine the accurate pathophysiology of cardiac valve disease in patients with Hunter syndrome.

## References

[1] Antoniou T, Kirvassilis G, Tsourelis L, Ieromonachos C, Zarkalis D, Alivizatos P (2009). Mitral valve replacement and Hunter syndrome: case report. Heart Surg Forum.

[2] Bhattacharya K, Gibson SC, Pathi VL (2005). Mitral valve replacement for mitral stenosis secondary to Hunter’s syndrome. Ann Thorac Surg.

[3] Braunlin EA, Harmatz PR, Scarpa M, Furlanetto B, Kampmann C, Loehr JP, Ponder KP, Roberts WC, Rosenfeld HM, Giugliani R (2011). Cardiac disease in patients with mucopolysaccharidosis: presentation, diagnosis, and management. J Inherit Metab Dis.

[4] Fesslova V, Corti P, Sersale G, Rovelli A, Russo P, Mannarino S, Butera G, Parini R (2009). The natural course and the impact of therapies of cardiac involvement in the mucopolysaccharidoses. Cardiol Young.

[5] Muenzer J, Wraith JE, Beck M, Giugliani R, Harmatz P, Eng CM, Vellodi A, Martin R, Ramaswami U, Gucsavas-Calikoglu M, Vijayaraghavan S, Wendt S, Puga AC, Ulbrich B, Shinawi M, Cleary M, Piper D, Conway AM, Kimura A (2006). A phase II/III clinical study of enzyme replacement therapy with idursulfase in mucopolysaccharidosis II (Hunter syndrome). Genet Med.

[6] Muenzer J, Gucsavas-Calikoglu M, McCandless SE, Schuetz TJ, Kimura A (2007). A phase I/II clinical trial of enzyme replacement therapy in mucopolysaccharidosis II (Hunter syndrome). Mol Genet Metab.

[7] Muenzer J, Beck M, Eng CM, Escolar ML, Giugliani R, Guffon NH, Harmatz P, Kamin W, Kampmann C, Koseoglu ST, Link B, Martin RA, Molter DW, Munoz Rojas MV, Ogilvie JW, Parini R, Ramaswami U, Scarpa M, Schwartz IV, Wood RE, Wraith E (2009). Multidisciplinary management of Hunter syndrome. Pediatrics.

[8] Wraith JE, Beck M, Giugliani R, Clarke J, Martin R, Muenzer J, Investigators HOS (2008). Initial report from the Hunter Outcome Survey. Genet Med.

[9] Wraith JE, Scarpa M, Beck M, Bodamer OA, De Meirleir L, Guffon N, Meldgaard Lund A, Malm G, Van der Ploeg AT, Zeman J (2008). Mucopolysaccharidosis type II (Hunter syndrome): a clinical review and recommendations for treatment in the era of enzyme replacement therapy. Eur J Pediatr.

